# Prognostic Analysis for Cardiogenic Shock in Patients with Acute Myocardial Infarction Receiving Percutaneous Coronary Intervention

**DOI:** 10.1155/2017/8530539

**Published:** 2017-01-30

**Authors:** Mao-Jen Lin, Chun-Yu Chen, Hau-De Lin, Han-Ping Wu

**Affiliations:** ^1^The Division of Cardiology, Department of Medicine, Taichung Tzu Chi Hospital, The Buddhist Tzu Chi Medical Foundation, Taichung, Taiwan; ^2^Department of Medicine, School of Medicine, Tzu Chi University, Hualien, Taiwan; ^3^Division of Emergency Medicine, Department of Pediatrics, Changhua Christian Hospital, Changhua, Taiwan; ^4^School of Medicine, Kaohsiung Medical University, Kaohsiung, Taiwan; ^5^Division of Pediatric General Medicine, Department of Pediatrics, Chang Gung Memorial Hospital at Linko, Kweishan, Taoyuan, Taiwan; ^6^College of Medicine, Chang Gung University, Taoyuan, Taiwan

## Abstract

Cardiogenic shock (CS) is uncommon in patients suffering from acute myocardial infarction (AMI). Long-term outcome and adverse predictors for outcomes in AMI patients with CS receiving percutaneous coronary interventions (PCI) are unclear. A total of 482 AMI patients who received PCI were collected, including 53 CS and 429 non-CS. Predictors for AMI patients with CS including recurrent MI, cardiovascular (CV) mortality, all-cause mortality, and repeated-PCI were analyzed. The CS group had a lower central systolic pressure and central diastolic pressure (both *P* < 0.001). AMI patients with hypertension history were less prone to develop CS (*P* < 0.001). Calcium channel blockers and statins were less frequently used by the CS group than the non-CS group (both *P* < 0.05) after discharge. Synergy between Percutaneous Coronary Intervention with Taxus and Cardiac Surgery (SYNTAX) score, CV mortality, and all-cause mortality were higher in the CS group than the non-CS group (all *P* < 0.005). For patients with CS, stroke history was a predictor of recurrent MI (*P* = 0.036). CS, age, SYNTAX score, and diabetes were predictors of CV mortality (all *P* < 0.05). CS, age, SYNTAX score, and stroke history were predictors for all-cause mortality (all *P* < 0.05). CS, age, and current smoking were predictors for repeated-PCI (all *P* < 0.05).

## 1. Introduction

Cardiogenic shock (CS) is uncommon in patients with acute myocardial infarction (AMI). However, an AMI complicated by CS is a complex syndrome which may induce low cardiac output and hypotension resulting in multiorgan dysfunction and mortality. Even with the introduction of modern intensive care units (ICUs), advanced medical treatment, and invasive devices, the short-term mortality and morbidity of AMI complicated by CS remain high [1–4]. The mortality rate for AMI complicated by CS after early revascularization, including percutaneous coronary intervention (PCI), is approximately 40% to 60%. In addition, as for age and gender, patients with AMI complicated by CS who are older than 75 years of age may have a higher one-year mortality than their younger counterparts [5, 6]. In addition, compared with men, women suffering from STEMI more often have concurrent CS, according to some studies [7].

In terms of an invasive strategy for CS, there was no difference in 30-day survival rate between PCI and coronary artery bypass graft (CABG) group [8]. On the other hand, the outcome data comparing multivessel with culprit lesion PCI is controversial. Thus, the best revascularization strategy for CS patients remains obscure [9–12].

Long-term prognosis of patients with AMI complicated by CS is still unclear and analysis of predictors for adverse clinical outcomes has not been well studied. Therefore, this study aimed to survey the clinical features and outcomes of patients with AMI complicated by CS compared to those without CS. Moreover, the risk factors for recurrent myocardial infarction (MI), cardiovascular (CV) mortality, all-cause mortality, and repeated-PCI in patients with AMI complicated by CS were analyzed.

## 2. Methods

### 2.1. Study Population

A retrospective survey of a prospective database was conducted via medical record review over a period from 2007 through 2014. AMI patients between 20 and 90 years of age were consecutively recruited from the inpatient clinic at Taichung Tzu Chi Hospital, Taiwan. They were divided into two groups: patients with AMI complicated by CS (the CS group) and AMI patients without CS (non-CS group). Patients suffering from out of hospital death (OHCA) and patients with malignancy were excluded from the analysis. All patients were followed up regularly via the outpatient department (OPD). A survey focusing on MI, repeated-PCI, CV mortality, and all-cause mortality was completed for each patient at the end of the study.

### 2.2. Definition, Data Collection, and Measurement

CS was defined as systemic blood pressure (BP) less than 80/50 mmHg or less than 90/60 mmHg after vasopressor therapy during admission to the emergency department. Diabetes was defined as a fasting plasma glucose level of more than 126 mg/dL, a casual plasma glucose level greater than 200 mg/dL, or a hemoglobin A1c (HbA1c) level of more than 6.5%. Hypercholesterolemia was defined as a serum cholesterol level of more than 200 mg/dL or an LDL-C level of more than 100 mg/dL. Chronic kidney disease (CKD) was defined as an estimated glomerular filtration rate (eGFR) of less than 60 mL/min/1.73 m^2^, which was equal to or more than stage III chronic kidney disease (CKD). Previous MI history was defined as a history of MI prior to admission, accompanied by a threefold elevation of cardiac enzymes from the baseline value.

Measurements of body parameters included body height, body weight, and body mass index (BMI). Baseline biochemical data collected on admission included fasting plasma glucose, serum creatinine, total cholesterol, high-density lipoprotein-cholesterol (HDL-C), low-density lipoprotein-cholesterol (LDL-C), and serum triglyceride level. As for the hemodynamic data, central aorta systolic pressure and central aorta diastolic pressure during cardiac catheterization were also collected. The central aortic pressure (CAP) was measured via a pigtail catheter while performing a coronary angiography. The angiographic findings included number and distribution of diseased vessels, number of treated vessels, and number of lesions. Left ventricular systolic function was usually calculated via two-dimensional echocardiography. Lesion severity and complexity were evaluated using the Synergy between Percutaneous Coronary Intervention with Taxus and Cardiac Surgery (SYNTAX) score. Related clinical parameters including baseline characteristics, related risk factors, hemodynamic data, angiographic findings, and treatment strategies such as drug medications after discharge or invasive procedures (balloon angioplasty, bare-metal stent deployment, or drug-eluting stent deployment) were compared between patients with CS and those without CS. In addition, this study attempted to identify the significant predictor of AMI patients developing cardiogenic shock and to analyze the adverse predictors of recurrent MI, CV mortality, all-cause mortality, and repeated-PCI procedures in patients with CS.

### 2.3. Statistical Analysis

The patients were divided into two groups: patients with AMI complicated by CS (the CS group) and AMI patients without CS (non-CS group). The independent *t*-test, chi-squared test, Fisher's exact test, and multivariate logistic regression analysis were used to compare the differences between the two groups. The log rank test and Kaplan-Meier curves were used for the survival analysis. The Cox proportional hazards model was used to test the effects of independent variables on hazards. *P* values less than 0.05 were considered significant. All analyses were performed using the statistical package SPSS for Windows (Version 23.0, SPSS Inc., Chicago, IL).

## 3. Results

A total of 482 patients who suffered from AMI were collected during the study period. Among them, there were 53 patients with AMI complicated by CS on admission, while 429 patients had no CS during admission. The mean age in the CS group compared with the non-CS group was 62.5 ± 12.1 years versus 63.6 ± 13.1 years, respectively (*P* = 0.573). The mean follow-up period was 94.4 ± 97.9 weeks for the CS group and 152.2 ± 108.4 weeks for the non-CS group.

Baseline clinical characteristics are shown in Table 1. Concerning the hemodynamic parameters, after inotropic agents and intra-aortic balloon pumping (IABP) usage, the CS group had a lower central systolic pressure (CSP) (110.8 ± 26.7 versus 132.4 ± 23.6 mmHg, *P* < 0.001) and a lower central diastolic pressure (CDP) (63.1 ± 17.6 versus 72.2 ± 13.2 mmHg, *P* < 0.001) compared with the non-CS group. As for the baseline biochemistry, there was no significant difference between the two groups.

The demographic data of the study population are shown in Table 2. ST-elevation myocardial infarction (STEMI) was more prevalent in the CS group compared with the non-CS group, and non-ST-elevation myocardial infarction was less prevalent in the CS group than in the non-CS group (*P* = 0.001). On the other hand, a history of hypertension was less prevalent in the CS group compared with the non-CS group (*P* < 0.001). In addition, patients in the CS group used P2Y12 receptor inhibitor of platelet (P2Y12 inhibitors) more frequently than those in the non-CS group (*P* = 0.04). By contrast, CCB and statins were less frequently used by the CS group compared with the non-CS group (*P* = 0.012 and *P* = 0.04, resp.).

The angiographic findings and clinical outcomes are shown in Table 3. The distributions of diseased vessels in the CS group compared with the non-CS group were single vessel disease, 34.0% versus 39.2%; dual vessel disease, 30.2% versus 35.2%; and triple vessel disease, 35.8% versus 25.6% (*P* = 0.285). The SYNTAX score was higher in the CS group than in the non-CS group (17.3 ± 10.4 versus 13.1 ± 8.0, *P* < 0.001).

**Figure 1:**
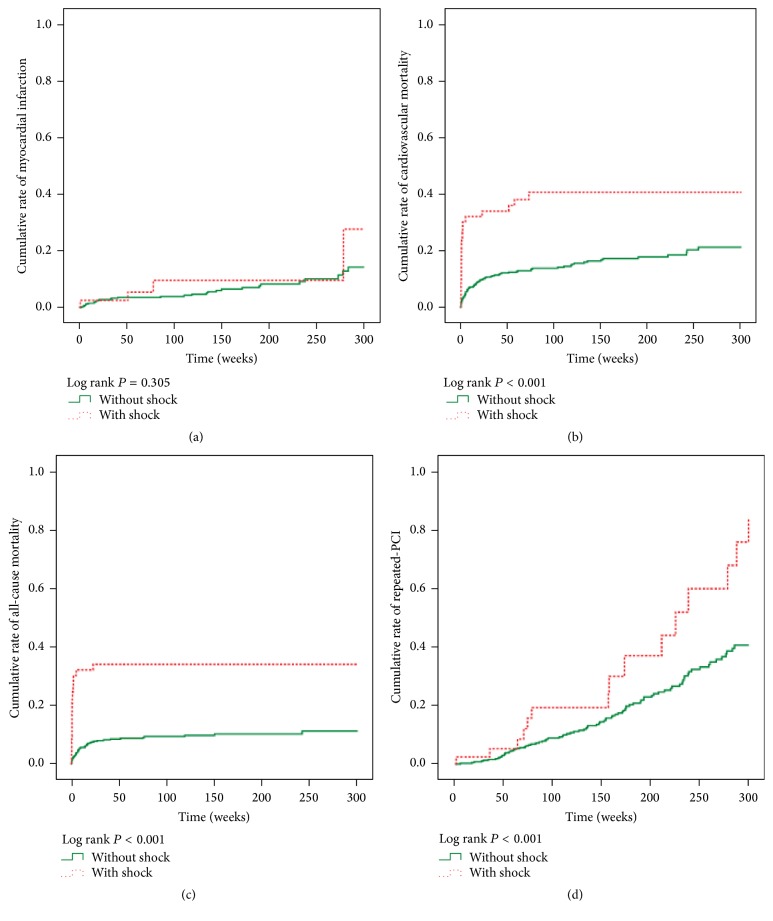
(a) Cumulative rate of myocardial infarction between the two groups (*P* = 0.305). (b) Cumulative rate of cardiovascular mortality between the two groups (*P* < 0.001). (c) Cumulative rate of all-cause mortality between the two groups (*P* < 0.001). (d) Cumulative rate of repeated-PCI between the two groups (*P* = 0.001).

Figure 1 shows the cumulative rate of freedom from recurrent MI, CV mortality, all-cause mortality, and repeated-PCI between the two groups. Freedom from CV mortality, all-cause mortality, and repeated-PCI was lower in the CS group compared with the non-CS group (all *P* < 0.001, resp.), but there was no significant difference between the two groups for recurrent MI (*P* = 0.305).

Medical factors predicting AMI complicated by CS are shown in Table 4. Based on the results of multivariate logistic regression analysis, SYNTAX score was the only predictor of AMI complicated by CS (*P* = 0.002). Furthermore, adverse predictors associated with clinical outcome in patients with AMI complicated by CS are listed in Table 5. For the CS group, history of stroke was a predictor of recurrent MI (*P* = 0.036), and CS, age, SYNTAX score, and diabetes were associated with CV mortality (*P* < 0.001, *P* < 0.001, *P* = 0.008, and *P* = 0.047, resp.). On the other hand, use of beta-blockers (BBs) and angiotensin-converting enzyme inhibitor (ACEIs) could reduce CV mortality. Moreover, CS, age, SYNTAX score, and history of stroke were associated with all-cause mortality (*P* < 0.001, *P* < 0.001, *P* = 0.002, and *P* = 0.003, resp.), whereas use of BBs and statins could reduce all-cause mortality. For repeated-PCI, CS, age, and current smoking were related risk factors (*P* < 0.001, *P* = 0.018, and *P* = 0.027, resp.), whereas usage of ACEIs could reduce the rate of repeated-PCI.

## 4. Discussion

AMI complicated by CS is one of the leading causes of death in patients hospitalized with AMI. Despite relevant progress even after invasive strategy, prognosis in this population remains poor and risk of future cardiac events is high. In this study, long-term CV mortality, all-cause mortality, and rate of repeated-PCI were higher in the CS group compared with the non-CS group. However, there was no difference between groups regarding recurrent MI. In addition, we found that SYNTAX score was an important risk factor for patients with AMI complicated by CS.

In our study, we also found that both CSP and CDP were lower in the CS group compared with the non-CS group in spite of use of inotropic agents or/and intra-aortic balloon pump (IABP). This finding was compatible with the reduced use of potent hypotensive agents such as CCB and angiotensin receptor blockers (ARB) in the CS group. Moreover, we found that hypertension was more prevalent in the non-CS group compared with the CS group.

The role of hypertension in AMI patients developing CS remains controversial. In a large observational study, the presence of hypertension in AMI patients may protect against developing CS [13], but, according to Menon and colleagues in the Global Utilization of Streptokinase and t-PA for Occluded Coronary Arteries (GUSTO) III Trial, hypertension was a predictor for developing CS in AMI patients [14]. There was no difference in terms of number or distribution of disease vessels from angiographic findings, but the left anterior descending artery (LAD) was the most common location of lesions in patients with AMI complicated by CS.

As has been reported, STEMI occurred more frequently in the CS group, but our CS group had higher SYNTAX scores than our non-CS group, which indicated that they had more complex and more severe lesion anatomy. Simple infarct-related artery (IRA) intervention in the CS group due to STEMI may have led to inadequate revascularization in these cases which could have affected long-term mortality and repeat-PCI rate. Immediate multivessel revascularization may be helpful in patients with CS due to STEMI [9] but whether this applies to all patients with AMI complicated by CS is still controversial [10].

The 30-day predictors for clinical outcomes of CS such as CS itself, DM, hypertension, previous MI, and old age have been previously studied [15]. From the results of logistic regression analysis, SYNTAX score was the only factor strongly related to developing CS in the AMI patients in our study. On the other hand, based on the results of our Cox proportional hazards model, CS, age, and SYNTAX score were risk factors associated with both long-term CV mortality and all-cause mortality. In patients with acute coronary syndrome (ACS), SYNTAX score was a significant predictor of both short-term and long-term outcomes. For STEMI patients receiving primary PCI, SYNTAX score was an independent predictor of short-term [16, 17] and long-term cardiac mortality [18, 19]. For NSTEMI patients receiving primary PCI, SYNTAX score was also an independent predictor of 1-year major adverse cardiac events (MACE) including death, cardiac death, MI, and target vessel revascularization (TVR) [20]. Clinically, SYNTAX score should be carefully evaluated during the index catheterization. AMI patients with high SYNTAX score deserve more attention, and more aggressive revascularization should be considered in these patients.

## 5. Conclusion

Patients with AMI complicated by CS may have higher long-term mortality rates and higher repeated-PCI rates than those without CS, but we found no significant difference in the occurrence of recurrent MI between the two groups. SYNTAX Score strongly correlated with development of CS in AMI patients during initial admission and may also predict long-term mortality in AMI patients with CS.

## Figures and Tables

**Table 1:** General characteristics of the study population.

Characteristics	Shock	Without shock	*P* value
Patient number	*N* = 53	*N* = 429	
Age (years)^a^	62.5 ± 12.1	63.6 ± 13.1	0.573
Height (cm)	1.6 ± 0.1	1.6 ± 0.1	0.685
Weight (kg)	67.3 ± 14.9	66.8 ± 13.2	0.822
BMI (kg/m^2^)	25.1 ± 4.5	25.1 ± 3.9	0.986
CSP (mmHg)	110.8 ± 26.7	132.4 ± 23.6	<0.001^*∗*^
CDP (mmHg)	63.1 ± 17.6	72.2 ± 13.2	<0.001^*∗*^
Glucose (mg/dL)	169.8 ± 94.6	149.9 ± 76.7	0.084
Cholesterol (mg/dL)	169.9 ± 55.8	179.2 ± 45.1	0.168
HDL (mg/dL)	39.6 ± 19.6	39.5 ± 15.9	0.946
LDL (mg/dL)	103.6 ± 43.4	111.7 ± 38.8	0.158
TG (mg/dL)	133.3 ± 94.1	140.3 ± 83.6	0.569
Serum creatinine (mg/dL)	1.7 ± 1.4	1.8 ± 2.2	0.793

BMI: body mass index; CSP: central aortic systolic pressure; CDP: central aortic diastolic pressure; HDL: high-density lipoprotein; LDL: low-density lipoprotein; TG: triglyceride. ^*∗*^Significant.

^a^Mean standard deviation.

**Table 2:** Demography of study population and medications during admission in patients with and without shock.

Characteristics	Shock (%)	Without shock (%)	*P* value
Gender			0.996
Male	41 (77.4)	332 (77.4)	
Female	12 (22.6)	97 (22.6)	
STEMI			0.001^*∗*^
Yes	36 (67.9)	184 (42.9)	
No	17 (32.1)	245 (57.1)	
Diabetes			0.597
Yes	23 (43.4)	170 (39.6)	
No	30 (56.6)	259 (60.4)	
Hypertension			<0.001^*∗*^
Yes	13 (24.5)	225 (52.4)	
No	40 (75.5)	204 (47.6)	
CKD			0.662
Yes	28 (52.8)	213 (49.7)	
No	25 (47.2)	216 (50.3)	
Hypercholesterolemia			0.125
Yes	22 (41.5)	226 (52.7)	
No	31 (58.5)	203 (47.3)	
Current smoker			0.981
Yes	23 (43.4)	185 (43.2)	
No	30 (56.6)	243 (56.8)	
Stroke history			0.673
Yes	4 (7.5)	26 (6.1)	
No	49 (92.5)	403 (93.9)	
CABG history			0.369
Yes	1 (1.9)	3 (0.7)	
No	52 (98.1)	426 (99.3)	
Aspirin			0.559
Yes	50 (94.3)	395 (92.1)	
No	3 (5.7)	34 (7.9)	
P2Y12 inhibitors			0.040^*∗*^
Yes	53 (100)	397 (92.5)	
No	0	32 (7.5)	
Diuretics			0.226
Yes	10 (18.9)	114 (26.6)	
No	43 (81.1)	315 (73.4)	
Beta-blockers			0.549
Yes	24 (45.3)	213 (49.7)	
No	29 (54.7)	216 (50.3)	
CCB			0.012^*∗*^
Yes	3 (5.7)	85 (19.8)	
No	50 (94.3)	344 (80.2)	
ACEI			0.247
Yes	15 (28.3)	156 (36.4)	
No	38 (71.7)	273 (63.6)	
ARB			0.090
Yes	5 (9.4)	81 (18.9)	
No	48 (90.6)	348 (81.1)	
Statins			0.040^*∗*^
Yes	14 (26.4)	176 (41.0)	
No	39 (73.6)	253 (59.0)	
Fibrate			0.845
Yes	2 (3.8)	14 (3.3)	
No	51 (96.2)	415 (96.7)	

Previous MI: history of previous myocardial infarction; CABG history: history of coronary artery bypass graft; CKD: chronic kidney disease; P2Y12 inhibitor: P2Y12 receptor inhibitor of platelet; CCB: calcium channel blocker; ACEI: angiotensin-converting enzyme inhibitor; ARB: angiotensin receptor blocker. ^*∗*^Significant.

**Table 3:** Demography of angiographic findings and clinical outcome.

Characteristics	Shock (%)	Without shock (%)	*P* value
Follow-up time (weeks)^a^	94.4 ± 97.9	152.2 ± 108.4	<0.001
Number of diseased vessel			0.285
Single vessel disease	18 (34.0)	168 (39.2)	
Dual vessel disease	16 (30.2)	151 (35.2)	
Triple vessel disease	19 (35.8)	110 (25.6)	
Mean of treated vessels	1.2 ± 0.5	1.2 ± 0.5	0.612
Mean of treated lesions	1.5 ± 0.7	1.5 ± 0.8	0.805
Lesion location			
LAD	42 (79.2)	335 (78.1)	0.847
LCX	31 (58.5)	237 (55.2)	0.654
RCA	35 (66.0)	232 (54.1)	0.098
SYNTAX score	17.3 ± 10.4	13.1 ± 8.0	<0.001
LVEF	0.5 ± 0.1	0.5 ± 0.1	0.387
Type of intervention			
Balloon angioplasty	12 (22.6)	128 (29.8)	0.276
BMS deployment	28 (52.8)	200 (46.6)	0.393
DES deployment	16 (30.2)	155 (36.1)	0.394
RMI			0.657
Yes	5 (9.4)	33 (7.7)	
No	48 (90.6)	396 (92.3)	
CV death			<0.001
Yes	18 (34.0)	43 (10.0)	
No	35 (66.0)	386 (90.0)	
All-cause death			<0.001
Yes	21 (39.6)	74 (17.2)	
No	32 (60.4)	355 (82.8)	
Re-PCI			0.501
Yes	16 (30.2)	111 (25.9)	
No	37 (69.8)	318 (74.1)	

LAD: left anterior descending artery; Lcx: left circumflex artery; RCA: right coronary artery; SYNTAX score: Synergy between Percutaneous Coronary Intervention with Taxus and Cardiac Surgery score; LVEF: left ventricular ejection fraction; BMS: bare-metal stent; DES: drug-eluting stent; RMI: recurrent myocardial infarction; CV death: cardiovascular death; Re-PCI: repeated percutaneous coronary intervention. ^a^Median (maximum-minimum).

**Table 4:** Significant predictors of CS for AMI patients in stepwise multiple logistic regression.

Variable	Adjusted OR	95% CI	*P* value
Age	0.99	0.96–1.01	0.373
Male	1.10	0.49–2.44	0.825
SYNTAX score	1.05	1.02–1.09	0.002^*∗*^
Smoke	0.84	0.42–1.67	0.615
Comorbidity			
STEMI	2.05	0.06–70.76	0.691
Non-STEMI	0.69	0.02–23.97	0.838
Dyslipidemia	0.77	0.40–1.51	0.447
Stroke	1.07	0.33–3.42	0.914
Diabetes mellitus	1.04	0.55–1.95	0.906
Medications			
Aspirin	1.48	0.41–5.41	0.549
Diuretics	0.69	0.31–1.54	0.366
BB	0.88	0.47–1.63	0.681
ACEI	0.55	0.27–1.12	0.098
Statin	0.49	0.23–1.02	0.056

Clinical factor used in analysis included sex, baseline biochemical data, angiographic findings on cardiac catheterization, exposed risk factors, and medications during admission. STEMI: ST-segment elevation myocardial infarction; Non-STEMI: non-ST-segment elevation myocardial infarction; BB: beta-blockers; ACEI: angiotensin-converting enzyme inhibitors. ^*∗*^Significant.

**Table 5:** Cox proportional hazard ratio of recurrent myocardial infarction, cardiovascular mortality, all-cause mortality, and repeated-PCI in AMI patients with cardiogenic shock after index PCI.

Variable	Recurrent MI		CV mortality		All-cause mortality		Repeat-PCI	
	Adjusted HR	*P* value	Adjusted HR	*P* value	Adjusted HR	*P* value	Adjusted HR	*P* value
With CS	1.57	0.442	4.51	<0.001^*∗*^	3.66	<0.001^*∗*^	2.93	<0.001^*∗*^
Age	1.03	0.094	1.06	<0.001^*∗*^	1.06	<0.001^*∗*^	1.02	0.018^*∗*^
Male	1.42	0.424	0.74	0.332	1.03	0.904	1.08	0.798
SYNTAX score	1.03	0.163	1.03	0.008^*∗*^	1.03	0.002^*∗*^	0.98	0.078
Comorbidity								
STEMI	2.53	0.870	2.85	0.308	3.16	0.251	5.02	0.740
Non-STEMI	5.78	0.758	3.28	0.258	4.72	0.128	6.20	0.707
DM	1.89	0.087	1.76	0.047^*∗*^	1.46	0.088	1.54	0.043
Dyslipidemia	0.91	0.817	0.91	0.737	0.98	0.931	0.87	0.540
Smoke	0.63	0.273	1.13	0.724	0.80	0.403	1.67	0.027^*∗*^
Stroke	3.19	0.036^*∗*^	2.04	0.075	2.53	0.003^*∗*^	1.09	0.873
Medications								
Aspirin	0.86	0.814	1.42	0.463	0.60	0.068	1.98	0.191
P2Y12 inhibitors	0.84	0.778	2.03	0.336	0.90	0.790	1.51	0.384
Diuretics	1.33	0.466	1.09	0.793	1.02	0.937	1.27	0.355
BB	0.83	0.610	0.42	0.005^*∗*^	0.50	0.004^*∗*^	1.02	0.938
ACEI	1.06	0.889	0.48	0.032^*∗*^	0.64	0.078	0.41	<0.001^*∗*^
Statin	0.67	0.379	0.57	0.129	0.50	0.023^*∗*^	0.93	0.784

Recurrent MI: recurrent myocardial infarction; CV mortality: cardiovascular mortality; CS: cardiogenic shock; SYNTAX score: Synergy between Percutaneous Coronary Intervention with Taxus and Cardiac Surgery score; DM: diabetes mellitus; STEMI: ST-segment elevation myocardial infarction; Non-STEMI: non- ST-segment elevation myocardial infarction; P2Y12 inhibitors: P2Y12 receptor inhibitor of platelet; BB: beta-blockers; ACEI: angiotensin-converting enzyme inhibitors ^*∗*^Significant.

## Abbreviations

ACEI: Angiotensin-converting enzyme inhibitor
AMI: Acute myocardial infarction
ARB: Angiotensin receptor blocker
BB: Beta-blockers
BMI: Body mass index
BMS: Bare-metal stent
CAD: Coronary artery disease
CABG: Coronary artery bypass graft
CCB: Calcium channel blockers
CKD: Chronic kidney disease
CDP: Central aortic diastolic pressure
CSP: Central aortic systolic pressure
CS: Cardiogenic shock
CV mortality: Cardiovascular mortality
DES: Drug-eluting stent
eGFR: Estimated glomerular filtration rate
FFR: Fraction flow reserve
HbA1c: Hemoglobin A1C
HDL-C: High-density lipoprotein-cholesterol
LAD: Left anterior descending artery
Lcx: Left circumflex artery
LDL-C: Low-density lipoprotein-cholesterol
LVEF: Left ventricular ejection fraction
OPD: Outpatient department
PCI: Percutaneous coronary intervention
RCA: Right coronary artery
SYNTAX score: Synergy between Percutaneous Coronary Intervention with Taxus and Cardiac Surgery score
TG: Triglyceride.

## Additional Points

*Study Limitations.* Our study had some limitations including geographical and country differences in invasive strategy which may have caused treatment bias and subsequent impact on outcomes. In addition, myocardial perfusion assessment such as Thrombolysis in Myocardial Infarction (TIMI) flow was not evaluated in this study. Thrombus aspiration and IABP insertion were also not routinely performed in this study, which may have had an impact on outcomes.

## References

[1] Kunadian V., Qiu W., Ludman P. (2014). Outcomes in patients with cardiogenic shock following percutaneous coronary intervention in the contemporary era: an analysis from the BCIS database (British Cardiovascular Intervention Society). *JACC: Cardiovascular Interventions*.

[2] Greenberg G., Assali A., Assa-Vaknin H. (2012). Outcome of patients presenting with ST elevation myocardial infarct and cardiogenic shock: a contemporary single center's experience. *Cardiology*.

[3] Aissaoui N., Puymirat E., Tabone X. (2012). Improved outcome of cardiogenic shock at the acute stage of myocardial infarction: a report from the USIK 1995, USIC 2000, and FAST-MI French Nationwide Registries. *European Heart Journal*.

[4] Sutton A. G. C., Finn P., Hall J. A., Harcombe A. A., Wright R. A., De Belder M. A. (2005). Predictors of outcome after percutaneous treatment for cardiogenic shock. *Heart*.

[5] De Felice F., Guerra E., Fiorilli R. (2014). One-year clinical outcome of elderly patients undergoing angioplasty for ST-elevation myocardial infarction complicated by cardiogenic shock: the importance of 3-vessel disease and final TIMI-3 flow grade. *Journal of Invasive Cardiology*.

[6] Prasad A., Lennon R. J., Rihal C. S., Berger P. B., Holmes D. R. (2004). Outcomes of elderly patients with cardiogenic shock treated with early percutaneous revascularization. *American Heart Journal*.

[7] Koeth O., Zahn R., Heer T. (2009). Gender differences in patients with acute ST-elevation myocardial infarction complicated by cardiogenic shock. *Clinical Research in Cardiology*.

[8] White H. D., Assmann S. F., Sanborn T. A. (2005). Comparison of percutaneous coronary intervention and coronary artery bypass grafting after acute myocardial infarction complicated by cardiogenic shock: results from the should we emergently revascularize occluded coronaries for cardiogenic shock (SHOCK) trial. *Circulation*.

[9] Park J. S., Cha K. S., Lee D. S. (2015). Culprit or multivessel revascularisation in ST-elevation myocardial infarction with cardiogenic shock. *Heart*.

[10] Zeymer U., Hochadel M., Thiele H. (2015). Immediate multivessel percutaneous coronary intervention versus culprit lesion intervention in patients with acute myocardial infarction complicated by cardiogenic shock: results of the ALKK-PCI registry. *EuroIntervention*.

[11] Mylotte D., Morice M.-C., Eltchaninoff H. (2013). Primary percutaneous coronary intervention in patients with acute myocardial infarction, resuscitated cardiac arrest, and cardiogenic shock: the role of primary multivessel revascularization. *JACC: Cardiovascular Interventions*.

[12] Thiele H., Desch S., Piek J. J. (2016). Multivessel versus culprit lesion only percutaneous revascularization plus potential staged revascularization in patients with acute myocardial infarction complicated by cardiogenic shock: design and rationale of CULPRIT-SHOCK trial. *American Heart Journal*.

[13] Ruiz-Bailén M., Rucabado-Aguilar L., Castillo-Rivera A. M. (2008). Cardiogenic shock in acute coronary syndrome in the Spanish population. *Medical Science Monitor*.

[14] Menon V., Hochman J. S., Stebbins A. (2000). Lack of progress in cardiogenic shock: lessons from the GUSTO trials. *European Heart Journal*.

[15] Singh M., White J., Hasdai D. (2007). long-term outcome and its predictors among patients with ST-segment elevation myocardial infarction complicated by shock. Insights from the GUSTO-I trial. *Journal of the American College of Cardiology*.

[16] Yang C.-H., Hsieh M.-J., Chen C.-C. (2013). The prognostic significance of SYNTAX score after early percutaneous transluminal coronary angioplasty for acute ST elevation myocardial infarction. *Heart Lung and Circulation*.

[17] Kul S., Akgul O., Uyarel H. (2012). High SYNTAX score predicts worse in-hospital clinical outcomes in patients undergoing primary angioplasty for acute myocardial infarction. *Coronary Artery Disease*.

[18] Magro M., Nauta S., Simsek C. (2011). Value of the SYNTAX score in patients treated by primary percutaneous coronary intervention for acute ST-elevation myocardial infarction: The MI SYNTAXscore study. *American Heart Journal*.

[19] Yang C.-H., Hsieh M.-J., Chen C.-C. (2012). SYNTAX score: an independent predictor of long-term cardiac mortality in patients with acute ST-elevation myocardial infarction. *Coronary Artery Disease*.

[20] Palmerini T., Genereux P., Caixeta A. (2011). Prognostic value of the SYNTAX score in patients with acute coronary syndromes undergoing percutaneous coronary intervention: analysis from the ACUITY (Acute Catheterization and Urgent Intervention Triage StrategY) trial. *Journal of the American College of Cardiology*.

